# Are we using drugs rationally? A survey study from Turkey

**DOI:** 10.12669/pjms.315.7370

**Published:** 2015

**Authors:** Serife Ozdinc, Nazli Sensoy, Rumeysa Kurt, Sevda Altas, Ramazan Altun

**Affiliations:** 1 Serife Ozdinc, MD. Assistant Professor, Department of Emergency Medicine, Afyon Kocatepe University, School of Medicine, Afyonkarahisar, Turkey; 2 Nazli Sensoy, MD. Associate Professor, Department of Family Medicine, Afyon Kocatepe University, School of Medicine, Afyonkarahisar, Turkey; 3 Rumeysa Kurt, 5th Grade Student, Afyon Kocatepe University, School of Medicine, Afyonkarahisar, Turkey; 4 Sevda Altas, 5th Grade Student, Afyon Kocatepe University, School of Medicine, Afyonkarahisar, Turkey; 5 Ramazan Altun, 5th Grade Student, Afyon Kocatepe University, School of Medicine, Afyonkarahisar, Turkey

**Keywords:** Rational use of drugs, Drug, Patient, Prescribed drug, Physician, Irrational use of drugs

## Abstract

**Objectives:**

To investigate the rational use of drugs from patient’s perspective.

**Methods:**

This study was conducted at the Afyon Kocatepe University Training and Research Hospital between February and March 2013. Data were collected with a questionnaire. Descriptive statistics and Chi-Square test were used.

**Results:**

About 54% (n=419) of participants reported that they used drugs without the advice of a physician. The 19-24 age group, secondary and high school graduates, and students used drugs more often without consulting a physician (P < 0.05). Participants that used drugs after consulting a physician did not fully use the drugs as recommended by the physician, and physicians did not give patients adequate information about prescribed drug(s). 72% of participants stored drug(s) at home.

**Conclusions:**

Rational use of drugs is not completely achieved. Certain patient groups and even physicians are closer to being a part of the irrational use of drugs.

## INTRODUCTION

Drugs, as technological products, are of great importance in public health in terms of not only the prevention of disease but also in fighting disease.^1^ Therefore, it is essential to ensure rational use of this valuable resource. Rational use of drugs (RUD) was defined by the World Health Organization (WHO) as “Patients receive medications appropriate to their clinical needs, in doses that meet their own individual requirements, for an adequate period of time, and at the lowest cost to them and their community”.^2^

Irrational use of drugs (IRUD) is the inability to fulfill one or several of these circumstances. Physicians, health care workers, patients, manufacturers and pharmacies create human sources of IRUD. The most important of these is the patients’ group. Therefore, it is desired that patients should participate in the treatment.^3^ Physicians should provide detailed information to patients about the prescription. Hence, patients are a substantial part of RUD.

Many studies that have been done to document drug use patterns indicate over prescribing, multi-drug prescribing, misuse of drugs, use of unnecessary expensive drugs and overuse of antibiotics and injections. In this study, we concentrated on the patients and aimed to investigate the RUD from the patient perspective.

## METHODS

This study was conducted between February and March 2013 at the Afyon Kocatepe University Training and Research Hospital (AKUTRH). The data of the study were collected by a questionnaire. The questionnaire consisted of 36 questions including 6 questions related to sociodemographic features and 30 questions about RUD. For questionnaire see Table-I. Total 774 patients who were admitted to the outpatient clinic of AKUTRH were included in this study. They were selected randomly.

**Table-I:** Questions summarized related to rational use of drugs.

Questions of questionnaire
Do you use drugs without physician advice if you feel ill?
If you use drugs without physician advice, how do you decide it?
What is your state of using drug(s) prescribed?
Do you use drug(s) in doses recommended by the physician? If yes, why?
Do you use drug(s) for the period recommended by the physician? If yes, why?
Do you consult a physician when the drug(s) side effect(s) occur(s)? If no, what do you do?
Does the physician explain the use of the drugs prescribed?
Do you look at the expiry date of the drug?
Do you read the information leaflet of the drug?
Have you written a prescription? If yes, does physician give you an information about your illness (diagnose, physical examination) and drug(s) prescribed (drug dose, drug duration, drug usage, drug side effect, warnings, drug–food interactions)?
Do you store drug(s) at home?
If yes, do you store analgesics/antibiotics/cold medications/ointments/antibiotics/digestive system drugs/vitamins and antiallergic drugs at home.

The questionnaire was filled after the patients had left the clinic. Before the questionnaire was applied to participants, they were told about the purpose of the study in order to eliminate participants’ concerns, and an informed consent form was read and signed. A preliminary questionnaire was conducted with 20 participants to evaluate the intelligibility of the questions in the questionnaire. It was filled out during face to face interviews. Unwilling and patients younger than 18 years of age were excluded.

The survey was approved by the Afyon Kocatepe University Ethics Committee (20 March 2013-94862910-33) and AKUTRH Chief of Staff. Analyses were made with the SPSS 18.0 (SPSS, Chicago, IL) package program. Both descriptive statistics and Chi-Square test were used. A p value below 0.05 was accepted as statistically significant.

## RESULTS

The mean age of the 774 participants in the study was 38±14, 55. 4% of them (n=429) were female. About 54% of participants (n=419) reported that they used a drug without consulting a physician when they had felt ill. Demographic factors affecting the use of drugs with or without a physician’s advice is given in Table-II. We found significant differences among participants in terms of age and education.

**Table-II:** Factors associated with participants who used the drug with/without consulting a physician.

	Group 1: participants who used the drug with consulting a physician			Group 2: participants who used the drug without consulting a physician			Statistics
	n	% of group 1	% of total	n	% of group 2	% of total	
*Age (years)*							
19-24	52	34.9	14.7	97	65.1	23.1	χ^2^=9.52
25-44	171	48.9	48.3	179	51.1	42.6	p= 0.023
45-64	108	46.6	30.5	124	53.4	29.5	
65+	23	53.5	6.5	20	46.5	4.8	
*Sex*							
Female	185	43.1	52.3	244	56.9	58.1	χ^2^=2.68,
Male	169	49.0	47.7	176	51.0	41.9	p> 0.05
*Marital status*							
Married	254	47.7	71.8	278	52.3	66.2	χ^2^=2.76,
Single	100	41.3	28.2	142	58.7	33.8	p> 0.05
*Education*							
Illiterate	25	64.1	7.1	14	35.9	3.3	χ^2^=19.64
Primary school	107	53.2	30.2	94	46.8	22.4	p= 0.0002
Secondary + high school	126	37.5	35.6	210	62.5	50	
University	96	48.5	27.1	102	51.5	24.3	
*Occupation*							
Housewife	128	46.2	36.2	149	53.8	35.5	χ^2^=7.5,
Unemployed + unworking	33	51.6	9.3	31	48.4	7.4	
Officer +retired	77	53.1	21.8	68	46.9	16.2	p> 0.05
Worker + artisan	52	40.9	14.7	75	59.1	17.9	
Student	64	39.8	18.1	97	60.2	23.1	
*Health insurance*							
Available	331	46.4	93.5	383	53.6	91.2	χ^2^=1.46,
Unavailable	23	38.3	6.5	37	61.7	8.8	p> 0.05

Two hundred ten(50%) of participants who used drugs without consulting a physician said that they chose a drug relying on their previous experience, 111 (26.5%) said that they chose a drug after consulting their family members, friends, or neighbors, 66 (15.7%) said that they chose a drug after consulting a non-physician health care professional and 33 (7.9%) said that they chose a drug after consulting a pharmacist.

It was found that 90.7% (n=321) of participants who used drugs after consulting a physician used their drugs in doses recommended by the physician, 57.6% (n=204) of them used their drugs for the period suggested by the physician, and 62.7% (n=222) of them consulted with the physician when drug side effects developed. About 25.1% (n=89) of participants who used drugs with consulting a physician declared that the physician explained the use of the drugs prescribed. The reasons for using the drug at different doses by the participants were fear about development of side effects (38.2%), forgetfulness- boredom (29.4%), thinking that their drug did not work (17.6%), not being given enough information about the drug by the physician (9.8%) and other reasons (5%). The causes of early stoppage of drug use were thinking that their disease was healed (65.2%), concern for development of side effects of the drug (18.2%), forgetfulness- boredom (11.1%), thinking that their disease was not healed (4%) and not receiving enough information about the drug from the physician (1.5%). When drug side effects developed, participants who did not consult a physician declared that they stopped using their own drug (66.5%), or they tried to find their own solutions (11.1%), or they consulted a pharmacist (1.1%), or did nothing (21.3%). About 78.2% (n=277) looked at the expiry date of the drug and 58.5% (n=207) read the information leaflet of the drug.

35.7% (n=273) of the participants stated that drugs were prescribed by the physician for them before the survey was performed. Distribution of participants’ declaration about information given by the physicians concerning diagnosis, physical examination and treatment is shown in Table-III.

**Table-III:** Distribution of *participants’ declaration about information given by physicians concerning diagnosis and treatment.

	Information was given		Information was not given		Total	
Information subject	n	%	n	%	n	%
Diagnosis	251	91.9	22	8.1	273	100
Physical examination	256	93.7	17	6.3	273	100
*Treatment*						
Drug dose- duration	135	49.5	138	50.5	273	100
Drug usage (IV/IM/PO…)	172	63.0	101	37.0	273	100
Drug side effect	128	46.9	145	53.1	273	100
Warnings	132	48.4	141	51.6	273	100
Drug–food interactions	135	49.5	138	50.5	273	100

IV: intravenous, IM: intramuscular, PO: peroral,

* participants who drug was prescribed (n=273)

Five hundred fifty eight (72%) participants stored drug(s) at home. These drugs stored at home were analgesics [87.1% (n=486)], cold medications [59.3% (n=331)], ointments [40.5% (n=226)], antibiotics [33% (n=184)], digestive system drugs [32.3% (n=180)], vitamins [31.2% (n=174)] and antiallergic drugs [23.8% (n=133)]. Demographic features associated with participants who did/did not store drug(s) at home are given in Table-IV. There were significant differences among them in terms of sex and occupation.

**Table-IV:** Demographic features associated with participants who did/did not store drug(s) at home.

	Group 1: participants who stored drug(s) at home			Group 2: participants who did not store drug(s) at home			Statistics
	n	% of group 1	% of total	n	% of group 2	% of total	
*Age (years)*							
19-24	110	73.8	19.7	39	26.2	18.1	χ^2^=2.82,
25-44	255	72.9	45.7	95	27.1	44.0	p= > 0.05
45-64	159	68.5	28.5	73	31.5	33.8	
65+	34	79.1	6.1	9	20.9	4.2	
*Sex*							
Female	326	76.0	58.4	103	24	47.7	χ^2^=7.26,
Male	232	67.2	41.6	113	32.8	52.3	p= 0.007
*Marital status*							
Married	390	73.3	69.9	142	26.7	65.7	χ^2^=1.24
Single	168	69.4	30.1	74	30.6	34.3	p> 0.05
*Education*							
Illiterate	22	56.4	3.9	17	43.6	7.9	χ^2^=5.74,
Primary school	150	74.6	26.9	51	25.4	23.6	p> 0.05
Secondary + high school	240	71.4	43.0	96	28.6	44.4	
University	146	73.7	26.2	52	26.3	24.1	
*Occupation*							
Housewife	207	74.7	37.1	70	25.3	32.4	χ^2^=11.09,
Unemployed + unworking	35	54.7	6.3	29	45.3	13.4	p=0.026
Officer +retired	104	71.7	18.6	41	28.3	19.0	
Worker + artisan	95	74.8	17.0	32	25.2	14.8	
Student	117	72.7	21.0	44	27.3	20.4	
*Health insurance*							
Available	514	72.0	92.1	200	28.0	92.6	χ^2^=0.05,
Unavailable	44	73.3	7.4	16	26.7	7.4	p> 0.05

## DISCUSSION

According to the WHO definition of RUD, the principles of RUD can be identified as being based on a true diagnosis, choosing the appropriate drug, prescribing it at a suitable dose and in a suitable way within the scope of a treatment plan and using it for a suitable duration, monitoring the side effects and patient compliance, measuring drug interactions, and considering the effect and cost of treatment. During any illness, it is expected that people go to a physician. When patients use a drug(s) without consulting a physician, these principles will be completely trampled. Use of drugs without consulting a physician, in other words self medication (SM), is a big issue all around the world. Studies in Africa, Asia and Latin America have detected that up to 80% of illness episodes are SM.^4^ SM has also been noted in the United States of America and Europe.^5^ In our study, SM rate was 54%. The reasons for the different rates in studies of SM may be the different interpretation of RUD by consumers. For the consumers, RUD is based on their age, prior knowledge, habits, beliefs, education, occupation, economic conditions, information, and the communication skills and attitudes of the prescriber and dispenser.

Yapici et al. found that patients who were under 19 years old, high school graduates, students, workers and artisans mostly used drugs without physicians’ advice.^6^ In our study, the 19-24 age group, the secondary and high school graduates were more self-medicated than other age, occupation and education groups. Moreover, participants in our study chose the drug(s) relying on their previous experience and consulting their family members, friends, neighbors, non-physician health care professionals and pharmacists. The Loa study also indicated that patients bought their drugs based on their own and friends’ decisions.^4^

According to the WHO, 50% of patients fail to take all of their drugs correctly.^7^ Correct prescribing does not guarantee that drugs will be properly used. Treatment compliance of the patients ranged between 20% and 78%.^8^ Causes of non-adherence to prescriptions are beliefs about the efficacy of certain drugs, the patient’s assessment of a particular disease, the communication skills and attitudes of health workers, lack of clear objectives and/or instructions of the drug prescribed, and different patients’ perceptions of the severity of the risk and side effects of drugs. In our study, we found that the participants who used drugs after consulting a physician did not fully use their drugs as recommended by the physician in terms of doses, period, and consultation with the physician when drug side effects developed. The reasons for this were may be patients’ wrong beliefs about drug(s), an awareness about serious issue of drug use and insufficient explanations given by a physician.

Another issue related to drug use is the habit of checking the expiry date of the drug. This can result in harmful consequences for health. In parallel to our study, in studies from Turkey, it was found that a high proportion of participants (88.4%, 71.7%) looked at the expiry date of the drugs which was quite heartening.^9^,^10^

If the physician does not give enough explanation of drug(s), sometimes even do so, reading the information leaflet of the drug(s) may have negative effects on patients. For instance, patients may give up their prescribed drugs as they may evaluate information incorrectly. So IRUD can occur. The rate of information leaflet reading in the studies was found to be 73.3%^9^ and 83.6%.^11^ However in our study, this rate was 58.5%. Though this rate may seem low, whatever the reason for this, it doesn’t disappoint us because of the reasons explained above.

According to RUD criteria, physicians must give information to patients about their illness and the use of prescribed drugs.^12^,^13^ Moreover, at the end of the visit, physicians should repeat the information given to the patient. Then the patient can be corrected about misperceptions and patient’s concerns can be relieved. Research conducts in the Philippines, Ethiopia, Ghana, India, Kenya, and Papua New Guinea showed that consumers know very little about the drugs they used.^14^ According to Basaran and Akici, physicians didn’t give enough information to patients about their diagnose and drugs.^3^ In our study, we could say that physicians didn’t give to all patients adequate information about their diagnose and drug(s) prescribed. The high number of patients examined daily and the lack of time allotted per patient could be one of the reasons.

A result of IRUD is to store drugs at home, or vice versa. It is possible to encounter drug(s) which remain either completely or partially unused at a person’s home. Even a small pharmacy was opened with these drugs. A study revealed that one or more such drugs are taken in 80% of homes in Turkey.^15^ Analgesics, antipyretics, anti-inflammatory drugs; antibiotics constitute the largest part of these drugs. A study from Belgium showed that drugs were found in all the houses included in the study.^16^ In our study, we also detected that most of the participants stored drug(s) in their homes. When people feel ill, they can use these drugs irrationally. They may encounter dangerous drug reactions and even die. Besides, this can cause difficulties in diagnosing illnesses, prolongation of treatment, development of antibiotic resistance and also drug waste. Moreover, it can lead to poisoning of their children.

In a study conducted among members of the military showed that there were no significant differences among participants who stored drug(s) at home in terms of sex, age and marital status.^9^ But we found that female and housewife, officer-retired and students participants stored more drug(s) at home compared with males and other occupation groups. Based on these results, we can say that women, house wifes, officers, retired and students want to be able to reach drugs at any moment. Hence, they are very close to IRUD.

## CONCLUSION

This study reveals that RUD is not entirely achieved. Certain patient groups and even physicians are closer to being a part of the IRUD. However, this common problem can be solved. Patients, especially the above mentioned patients, can be informed and trained in detail about drugs and also about prescribed drugs. Moreover, physicians can be trained in giving information to patients. Trained physicians, mass media and social media can be used for public education and warning.
